# Stereological Changes in Microvascular Parameters in Hippocampus of a Transgenic Rat Model of Alzheimer’s Disease

**DOI:** 10.3233/JAD-210738

**Published:** 2021-10-26

**Authors:** Yaroslav Kolinko, Lucie Marsalova, Stephanie Proskauer Pena, Milena Kralickova, Peter R. Mouton

**Affiliations:** a Biomedical Center, Faculty of Medicine in Pilsen, Charles University, Pilsen, Czech Republic; b Department of Histology and Embryology, Faculty of Medicine in Pilsen, Charles University, Pilsen, Czech Republic; c SRC Biosciences, Tampa, FL, USA; d University of South Florida, Tampa, FL, USA

**Keywords:** Alzheimer’s disease, capillary, hippocampus, microvessels, stereology, TgF344-AD rat

## Abstract

**Background:**

Microcirculatory factors play an important role in amyloid-β (Aβ)-related neuropathology in Alzheimer’s disease (AD). Transgenic (Tg) rat models of mutant Aβ deposition can enhance our understanding of this microvascular pathology.

**Objective:**

Here we report stereology-based quantification and comparisons (between- and within-group) of microvessel length and number and associated parameters in hippocampal subregions in Tg model of AD in Fischer 344 rats and non-Tg littermates.

**Methods:**

Systematic-random samples of tissue sections were processed and laminin immunostained to visualize microvessels through the entire hippocampus in Tg and non-Tg rats. A computer-assisted stereology system was used to quantify microvessel parameters including total number, total length, and associated densities in dentate gyrus (DG) and cornu ammonis (CA) subregions.

**Results:**

Thin hair-like capillaries are common near Aβ plaques in hippocampal subregions of Tg rats. There are a 53% significant increase in average length per capillary across entire hippocampus (*p*≤0.04) in Tg compared to non-Tg rats; 49% reduction in capillary length in DG (*p*≤0.02); and, higher microvessel density in principal cell layers (*p*≤0.03). Furthermore, within-group comparisons confirm Tg but not non-Tg rats have significant increase in number density (*p*≤0.01) and potential diffusion distance (*p*≤0.04) of microvessels in principal cell layers of hippocampal subregions.

**Conclusion:**

We show the Tg deposition of human Aβ mutations in rats disrupts the wild-type microanatomy of hippocampal microvessels. Stereology-based microvascular parameters could promote the development of novel strategies for protection and the therapeutic management of AD.

## INTRODUCTION

Microvasculature disruptions in the brain parenchyma, especially in neocortical and hippocampal subregions, are part of the progressive neurodegeneration in brains of patients with Alzheimer’s disease (AD). These microvascular changes appear closely associated with neuroinflammation caused by microglia activation and release of pro-inflammatory cytokines in response to the deposition of insoluble amyloid-β (Aβ) protein [1]. Clinical studies and postmortem analysis suggest a significant interaction between Aβ deposition and microvascular pathology in a large majority (∼90%) of patients with confirmed AD [2–5].

The vascular development and anatomy of the rat hippocampus show strong similarities to those in humans. Arterial vascularization of the hippocampus emerges from the collateral branches of the posterior cerebral artery and the anterior choroidal artery, forming the network of superficial hippocampal arteries that branch into the deep intrahippocampal arteries with higher levels of vascularization in ventral as opposed to dorsal hippocampus [6]. Some evidence suggests the *stratum pyramidale* (pyramidal cells layer) of the Amun’s horns (CA) regions of hippocampus contain the highest density of the microvessels [6]. On the venous side, vascularization begins with the intrahippocampal veins that drain into the superficial hippocampal veins [7].

Previous studies of transgenic presenilin mouse models of familial AD report many similarities with the vascular pathology seen in patients with AD, including an age-related increase in the frequency of kinked, twisted, and string-like vessels [8]. Adult (7-month-old) double transgenic (APPswe/PS1delta E9) mice show a reduction in total number of capillary segments with no changes in capillary length in white matter (corpus callosum) [9]. These changes may reflect an adaptive response of the capillary network to the neuronal degeneration-related reduction in energy demands [10]. Other studies involving Aβ deposition in animal models and in cultured endothelial cells report variable degrees of vascular wall degeneration, loss of basal membrane and distorted, swollen nuclei in endothelial cells associated with the formation of micro-hematomas [8, 11, 12]. These small hemorrhages appear to result from Aβ deposition in the tunica media and smooth muscle cells in tunica adventitia [13, 14], leading to accelerated development and progression of amyloid angiopathy in cerebral capillaries [15]. These experimentally induced changes in vascular wall structure cause a generalized increased in the production of extracellular matrix-related proteins [15]. Microscopic analyses reveal a strong association between microvessel (capillary) length and the numbers of synaptic and neuronal mitochondria in the hippocampus [16, 17]. The pericytes that functionally regulate capillary contraction for the maintenance of blood follow [18–20] appear to undergo changes associated with vascular Aβ deposition [8, 21–25]. The deposition of mutant Aβ in the cell culture also appears to mediate neural stem cell maturation [26] and affect glial cells dysregulation [27]. Finally, antemortem functional magnetic resonance imaging and post-mortem studies of AD patients support the view that degenerative vascular changes lead to impaired vascular remodeling and/or angiogenesis [28, 29].

In the past two decades transgenic (Tg) mouse models of AD have shown the vascular consequences of expression of human AD mutants and deposition of Aβ peptides [8, 9, 23, 24, 26, 30]. The overexpression of familial AD-related mutations in rats [31–33] has further advantages due to the closer evolutionary relationship between humans and rats; and, the relative higher behavioral complexity of rats compared to mice [34]. Here we report on the TgF344-AD model that overexpresses the human APPswe and PS1*Δ*E9 mutation under the mouse prion protein promoter [21] in the Fischer 344 rat. These TgF344-AD rats show age-related cerebral amyloidosis prior to the progressive deposition of Aβ plaques, tauopathy, and neuronal loss leading to the manifestation of cognitive decline [32]. To date, however, only a few studies have focused on the microvascular bed in this and other rat Tg models of AD [35–40]. The present study is the first to assess capillary parameters using rigorous stereological methods to quantify differences in microvascular structure of Tg rats and non-Tg littermate (controls). We assessed between-group differences (Tg vs. non-Tg) and within-group differences for stereology parameters related to number and length of microvessels in hippocampal subregions, including total hippocampus (neuron cell layers, molecular layer, and white matter) and three hippocampal principal cells layers (PCL): 1) granule cell layer in dentate gyrus (DG); 2) pyramidal cell layer in CA3 combined with CA2 (CA 2/3); and 3) pyramidal cell layer in CA1. Second, we analyzed groups of Tg and non-Tg rats for within-group differences in microvascular parameters in these hippocampal subregions.

## MATERIAL AND METHODS

### Animals and ethical statements

Animals for this study were 12-month-old male rats from TgF344-AD (Tg) litters (*n* = 6) and non-Tg littermates (*n* = 5). The rats were housed in polysulfonate cages and maintained in a facility with 12-h light/dark photoperiod at a temperature of 21±1°C with a relative humidity of 60%. Standard commercial pellet diet and water were available *ad libitum*. All procedures were conducted in accordance with Act No. 246/1992 Coll. for the Protection of Animals against Cruelty under the supervision of the Animal Welfare Advisory Committee at the Ministry of Education, Youth and Sports of the Czech Republic (approval ID MSMT-11925/2016-3).

### PCR genotyping

To confirm genotype a sample (2 mm) of tissue was taken from the tail of each rat (Total *n* = 11) at the age of 8–10 weeks. Total purification of DNA was used according to the DNeasy Spin-Column Protocol (QIAGEN, Germany). The DNA of Tg rats included both APPsw and *Δ* exon 9 mutant human presenilin-1 (PS1*Δ*E9) genes while non-Tg rats were negative for either or both of these human mutations.

### Tissue processing

The animals were sacrificed by transcardial perfusion of 0.1 M phosphate buffered saline (PBS; pH 7.4) followed by 4% paraformaldehyde in PBS. Brains were immersion fixed in 70% alcohol for 24 h at 4°C then moved to 10% phosphate-buffered formalin for 30 days until embedding in paraffin blocks. Each block was microtome sectioned in the horizontal plane into 18-μm thick serial sections (∼350±16 sections per brain). Every 30th section was mounted to glass slides, immunostained using anti-laminin (see below) and counterstained with hematoxylin. An unbiased systematic-random sampling design was used to generate a set of about 8 to 11 sections depending on length of each hippocampus.

### Immunohistochemistry

Microvessels in the rat hippocampus were colorized using the polyclonal rabbit anti-laminin antibody (dilution 1:1000; Dako, Glostrup, Denmark, No. Z009701) with negative immunohistochemistry controls. Laminin is a marker for basal lamina present in all microvessels, including capillaries, venules, and arterioles. The sections were deparaffinized in xylene, rehydrated and successively treated with cooled acetone for 10 min; Proteinase K for 6 min at room temperature; 5% normal goat serum for 20 min at room temperature; primary antibody solution for 36 h at 4°C; 50% N-Histofine Simple Stain MAX PO (Multi, Nichirei Biosciences Inc., Tokyo, Japan) for 30 min at room temperature; and, colorized for 1–4 min in liquid diaminobenzidine (DAB) Substrate Chromogen System (Dako, DAB Chromogen). Immunostained sections were counterstained with Gill’s hematoxylin. In the final step, sections were dehydrated in an alcohol series cleared by xylene, treated with mounting medium and coverslipped.

### Quantitative processing

Stereological analyses were done blind to genotype with assistance from the Stereologer system (SRC Biosciences, Tampa, FL, USA) that consists of a Nikon Eclipse Ti-U microscope equipped with ProScan III motorized 3-axis step motor (Prior Electronics, UK) and standard optical lenses; high resolution digital imaging camera (Promicra 3-3CC); and the current version of Stereologer software (v11.0). As shown in Table 1, microvascular parameters included total region volume (Total V_Reg_); total number of microvascular segments (Total N_cap_); total number of microvascular endpoints (Total N_endp_); and total length of microvessels (Total L_cap_). These parameters were analyzed in the following reference volumes: total hippocampus (principal cell layers, molecular layer and white matter) and three hippocampal principal cell layers (PCL): granule cell layer in DG; pyramidal cell layer in CA3 combined with CA2 (CA 2/3); and pyramidal cell layer in CA1 [41, 42]. The regional volumes of each reference space were estimated using the Cavalieri principle with point counting [43, 44] with 10× objective (Plan Fluor, NA 0.45). Briefly, total volume of each region (Total V) was quantified from the sum of points (∑P) hitting each subregion using Total *V* = ∑P • area per point (μm) • sampling interval (k) • t, where t is the final post-processing section thickness (μm).

**Table 1:** Complete quantitative parameters of the microvascular network of hippocampus of non-Tg control (non Tg) and TgF344-AD rats (Tg). The stereological quantitative results are presented as the mean±standard deviation (SD)

			Volume (mm^3^) (mm^3^)	Volume fractions	Total capillary number	Total endpoints number	Capillary density (#/mm^3^)	Endpoints density (#/mm^3^)	Length (m)	Length density (m/mm^3^)	Mean Length (μm)	Diffusion distance (μm)
Total	non Tg	mean	46.40		1,228,668	622,140	27,147	13,742	75.99	1.67	62	7.19
		±SD	4.11		358,030	178,329	9,990	4,996	22.81	0.60	9	1.36
	Tg	mean	49.12		932,315	472,076	19,220	9,732	82.80	1.72	95*	7.01
		±SD	6.39		262,556	131,335	5,823	2,918	19.09	0.50	40	1.16
	non Tg	mean	2.00	4.37%	77,976	39,345	39,872	20,127	3.55	1.87	58	6.65
		±SD	0.56	1.41%	33,907	16,963	19,819	9,925	0.46	0.45	36	0.86
	Tg	mean	1.40	2.81%	90,070	45,691	62,976	32,049	4.49	3.03	59	5.62
CA 2/3
		±SD	0.45	0.77%	69,728	34,983	42,192	21,151	3.04	1.75	42	1.49
	non Tg	mean	1.56	3.36%	253,061	126,891	145,097	72,785	2.94	1.90	41	6.54
		±SD	0.23	0.38%	299,613	149,786	156,826	78,371	0.53	0.39	36	0.66
	Tg	mean	1.29	2.62%	28,355	19,218	22,542	15,292	3.08	2.48	111	5.74
CA 1
		±SD	0.44	0.75%	8,444	5,638	4,723	3,193	0.77	0.51	15	0.54
	non Tg	mean	2.05	4.43%	69,840	35,283	35,192	17,776	3.40	1.70	53	7.28
		±SD	0.30	0.68%	37,760	18,875	20,617	10,315	1.34	0.74	11	1.71
	Tg	mean	1.98	3.88%	45,521	23,127	21,255	10,887	1.72^*^	1.08	63	9.31
DG
		±SD	1.09	1.70%	34,532	17,241	8,197	4,164	0.29	0.49	48	2.67
	non Tg	mean	5.61	12.17%	148,864	400,877	27,060	74,552	9.88	1.78	68	6.76
		±SD	0.60	1.78%	39,724	254,667	8,621	51,663	1.51	0.37	11	0.69
	Tg	mean	4.67^†^	9.31% ^†^	163,946^†^	88,036^†^	35,911^†^	19,393^†^	9.30^†^	2.07	60	6.39
	PCL
		±SD	1.68	2.41%	70,666	35,259	13,883	6,857	3.36	0.67	11	0.96

*Significant in Mann-Whitney U Test; ^†^Significant in Friedman ANOVA analysis.

**Fig. 1:**
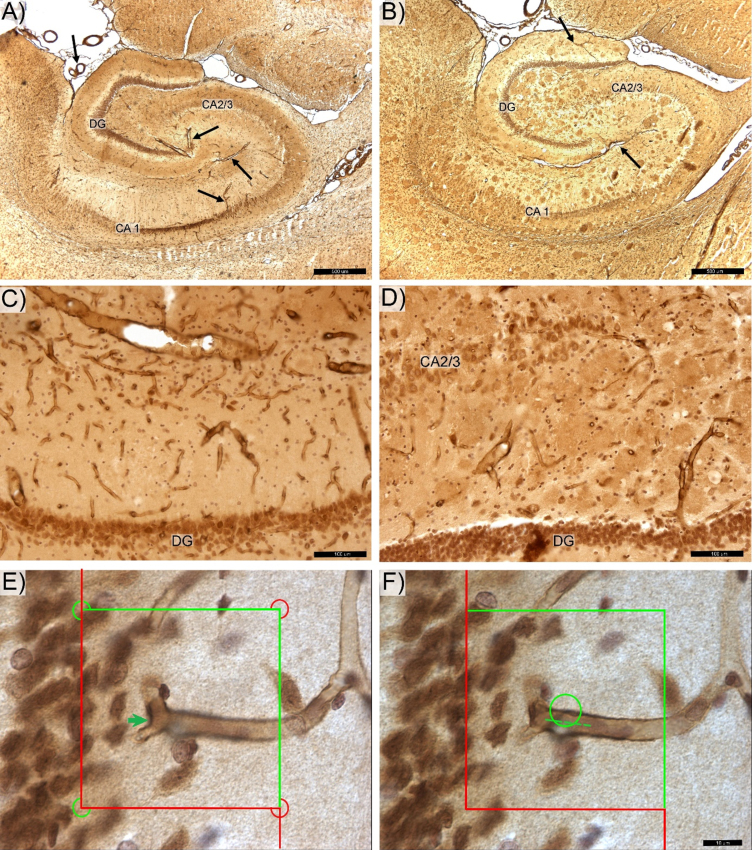
Photomicrograph of the anti-laminin antibody labeled hippocampal microvessels in control (A,C) and transgenic (B,D) rats. DG -, CA 2/3-, and CA1-labeled respective zone of the pyramidal cells layer. The magisterial vessels, serve as the main source of hippocampal blood supply (black arrows). E) Total number of capillary segments (Euler number) was quantified by counting “saddle points” (green arrow) using the 3D optical disector probe. F) Total capillary length was determined using the Space Balls method based on number of intersections between surface of the virtual 3D sphere probe and centerline (spline) of each capillary (dotted line). Space bars: A,B –500 μm; C,D –100 μm; E,F –20 μm.

A microvessel (capillary) was defined as a loop created between two vessel branches nodes (branch point or saddle point) of the vascular network [45, 46]. Total N_cap_, N_endp_, and L_cap_ were quantified using 60×oil objective (CFI, Plan Apo Lambda, NA 1.4) with 1-μm guard zones at the top and bottom surfaces of each section. The total N_cap_ and Total N_endp_ were calculated according to Gundersen’s Euler number based on the number of nodes (saddle points) counted by thin focal-plane z-axis scanning using the optical disector method [47, 48] (Fig. 1E). Number of Total N_cap_ was calculated as twice (2x) the number of nodes and the Total N_endp_ as the number of nodes +1 [9, 44, 49]. Estimates of total microvascular length (Total L_cap_) were made using the isotropic sphere probe (Space Balls) where the total number of sphere probe-capillary intersections (∑I) is directly proportional to the total microvascular length (Fig. 1F) [44, 50]. Average densities of microvascular number, microvascular branches, and microvascular length were calculated from Total N_cap_ and Total L_cap_, respectively, divided by the Total V of the respective reference space. Average microvascular length was calculated as the ratio of total microvascular length to total number of capillaries (Total L_cap_/ Total N_cap_). Finally, the potential diffusion distance of microvessels was determined in post-processing using the approach described in [46] with the gamma function calculated from average microvascular density. The coefficient of error (CE), a measure of sampling error, was estimated according to Gundersen et al., 1999 [51] with sampling across both hemispheres continued to a high level of sampling stringency [CE Total *V* = 0.03; CEs Total N_cap_ and Total L_cap_ = 0.13].

### Statistical analysis

We carried out non-parametric statistical comparison with assistance from Statistica 13 (StatSoft, Inc., Tulsa, OK, USA) to test for between-group (Tg versus non-Tg) and within-group (e.g., DG versus CA subregions in Tg or non-Tg rats) differences in Total V, Total N_cap_, or Total L_cap_ and the potential diffusion distance of microvessels in neuron cell layers of hippocampus (DG, CA1, and CA2/3). The Mann–Whitney U-test was used to test for group effects (Tg versus non-Tg). Within-group differences were assessed using Friedman ANOVA test followed by *post-hoc* testing using Wilcoxon signed-rank test. Correlations between parameters were quantified using the Spearman’s coefficient. Difference of *p* < 0.05 or lower was considered statistically significant.

## RESULTS

**Fig. 2:**
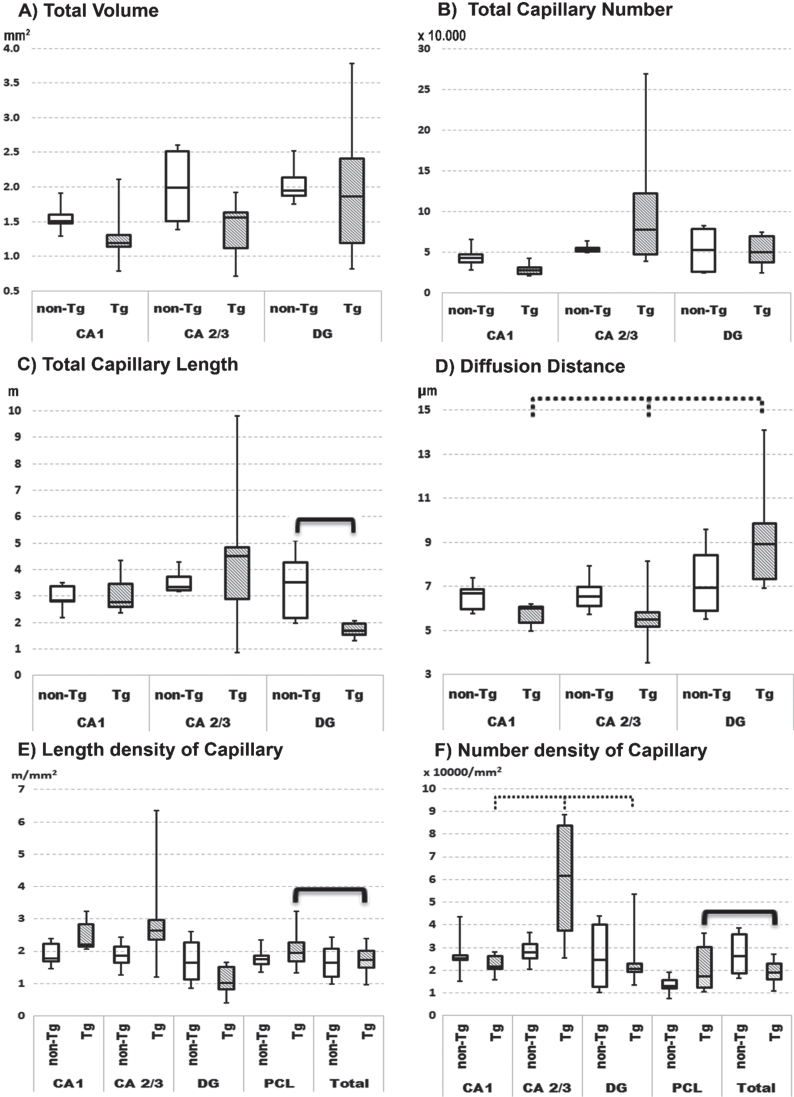
Quartile box plot of selected quantitative parameters for the microvascular network in anatomical subregions of pyramidal cell layer of hippocampus in non-transgenic (non-Tg) and transgenic rats (Tg). Significant results of the Mann–Whitney U-test are showed with bold lines (*p*≤0.05). Significant results of the Friedman ANOVA and Kendall coefficient of concordance within group are showed with bold dotted lines (*p*≤0.05). The stereological quantitative results are presented as median values with boxes that span the limits of the first and third quartiles and whiskers that span the minimum and maximum values for each group.

**Fig. 3:**
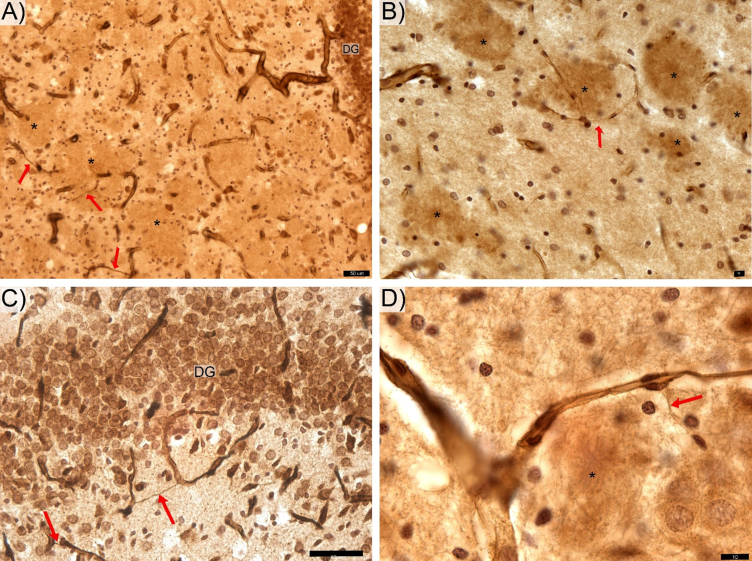
Features of thin hair-like capillaries (red arrows) commonly find in TgF rats. DG-, CA 2/3-, and CA1-labeled respective zone of the pyramidal cells layer. The anti-laminin antibody labeled hippocampal sections from the transgenic rats contain areas of with clear contours (*) appear as likely locations of Aβ deposition. Space bars: A,C – 50 μm; B, D – 10 μm.

Hippocampal sections from Tg rats show plaques with clear contours (Fig. 1B,D and Fig. 3) and prominent findings of abnormal hair-like capillaries in the vicinity of Aβ plaques (Fig. 3). Between-group comparisons (Tg versus non-Tg) of stereology parameters reveal a 49% reduction in Total L_cap_ in DG for Tg rats (Mann-Whitney U, *p*≤0.022; Fig. 2C). Furthermore, there is a 53% increase in average length per capillary in entire hippocampus including PCL and white matter in Tg rats (Mann-Whitney U, *p*≤0.036). There are no significant between-group differences in Total V, Total L_cap_ and Total N_cap_ for entire hippocampus (Mann-Whitney U, *p* > 0.05; see Table 1).

Tg rats show significant within-group differences in average N_cap_ density between PCL sublayers in Tg rats (Friedman, *p*≤0.011), with *post-hoc* confirmation of significantly higher average N_cap_ density in CA2/3 compared to CA1 (Wilcoxon, *p*≤0.027) and DG (Wilcoxon, *p*≤0.027). Second, Tg rats have significant within-group differences in the potential diffusion distance of microvessels (Friedman, *p*≤0.042), with *post-hoc* testing showing a significantly higher diffusion distance in DG comparing both CA2/3 and CA1 subregions (Wilcoxon, *p*≤0.046; Fig. 2). In contrast, non-Tg rats showed no significant within-group differences in the average densities of N_cap_ and L_cap_ or the potential diffusion distance of microvessels in PCL subregions (Fig. 2; Friedman, *p* > 0.05).

In Tg rats there are within-group differences in specific hippocampal subregions versus comparable subregions for the entire hippocampus. For instance, there is a significantly 56% higher N_cap_ density and 16% higher L_cap_ density in PCL as compared to the same parameters for the total hippocampus (Fig. 2 E, F; Wilcoxon, *p*≤0.046). In contrast, non-Tg rats show significant within-group differences for N_cap_ and L_cap_ densities (Wilcoxon, *p*≤0.05) in combined specific subregions as compared to the entire hippocampus.

## DISCUSSION

Blood vessels occupy about 8% of total volume of hippocampus in rats [52] with their spatial distributions as described earlier [6, 53]. From superficial hippocampal arteries these vessels form a network located for the most part in *stratum oriens* of the CA 1–3 zones and part of the molecular layer of DG zone (see Fig. 1A-C). Vessels extending from the inter-hippocampal artery in the hippocampal fissure provide blood to the internal part of the *molecular layer* of DG and the *stratums lacunosum, moleculare*, and *radiatum* of all CA subregions. The microvascular network of both pools then evenly spread along the large vessels and join in the PCL. Vascularization of PCL is characterized as deep microvascular network without preferential orientations [54], in contrast to white matter, with several large vessels that span all CA regions (Fig. 1) [6].

Previous studies in different cohorts of TgF344-AD rats found evidence of neurodegeneration [32, 55, 56]. Other studies in TgF344-AD rats reported electrophysiological changes starting [57, 58] and increase at glutamate receptors density in CA regions [59] at 6 months of age with no impairments in cognitive function until 10-12 months [32, 60]. Longer lived TgF344-AD rats after 12 months of age displayed pronounced deficits in memory and navigation [61–63] combine with progressive loss of hippocampal norepinephrine levels, especially in DG [60]. Our present study of male 12-month-old TgF344-AD rats found a significant 49% reduction in Total L_cap_ in DG with increases in mean diffusion distance of microvessels (Fig. 2). This finding can also be enhanced by slight decrease in N_cap_ and the volume of DG area. Because DG is a region with high neurogenic ability and neurogenic potential, we speculated that this finding provides an anatomical basis for reported evidence of neurodegeneration, electrophysiological disturbances, and cognitive decline [64] in TgF344-AD rats, especially in the early-stage of AD development.

Though the present study did not aim to stain the Aβ plaques, rings of microglial cells surrounding areas of with clear contours (Fig. 1B,D and Fig. 3) appear as likely locations of Aβ deposition [31, 32]. The generally non-uniform distribution of these apparent Aβ plaques in the hippocampus is similar to that reported by others [21, 32]. The area of hippocampus coverage by Aβ plaque gradually increasing from ∼0% in 6-month-old rats [32] to ∼3% in 12–17-month-old rats [21, 61]. Our findings of thin hair-like capillaries (Fig. 3) in proximity to apparent amyloid plaques in the hippocampus of this Tg rat model show similarities to those reported in the hippocampal microvascular system of Tg murine models of AD [8, 65]. These common observations in Tg rodent models of AD provides indirect evidence of the cerebral amyloid angiopathy found in hippocampal microcirculation of AD patients at postmortem examination [21, 65, 66].

A general trend in favor of decreasing volumes for all principal cells sublayers findings in our study (see Fig. 2A and Table 1) provide evidence of degeneration in Tg rats corresponds to that reported by other groups [31–33], including a denser capillary network in PCL comparing to the entire hippocampus of Tg rats. Furthermore, the average microvessels length in the denser microvascular network of hippocampus in Tg rats was twice that in the hippocampus of non-Tg rats. Among the possible explanations is microcirculatory remodeling in response to stress or injury caused by Aβ deposition in the hippocampus of Tg rats [67–69] to maintain the principal cells by limiting metabolic activity of white matter. The non-significantly higher average length of vessels in CA1 and DG areas combined with fluctuations of N_cap_ and L_cap_ in CA2/3 provide further support for this view.

We also found apparent discrepancies with earlier reports in Tg rodent models of AD. For example, previous stereology studies of microvascular in 7-month-old Tg APP/PS1 mice [9, 64] report reductions in the total N_cap_ in white matter and a 45% reduction in total L_cap_ up to 45% and reductions in hippocampal volume up to 30% [8]; in contrast, we did not find these effects in 12-month-old male TgF344-AD. Furthermore, we did not detect changes in mean L_cap_ density in the CA1 as reported in Flinders-sensitive line rats [67, 69, 70]. Possible reasons for these discrepancies include differences in the relative vulnerabilities of the microsvascular systems in hippocampus of mice and rats in response to Aβ deposition, as well as potential differences in the time course of these AD-type changes to manifest in different species of rodents. Further studies of the time course of these changes would help to clarify the similarities and differences of these changes in mice and rats in relation to similar changes in humans with AD.

Here we described previously unreported lower Total L_cap_ in DG of Tg rats, which may be the results of genetic features or possibly angiogenic responses to Aβ deposition. From about 9 months of age these vessel walls begin to lose their elasticity [71] and appear to undergo penetration by Aβ aggregates [18, 20, 21]. The cytoskeletal proteins desmin and α-smooth muscle actin (α-SMA) in pericytes become upregulated [21], which slows down the active redistribution of local blood flow [72]. Local diffusion changes could promote transient fluctuation of the blood pressure [71] that may induce or exacerbate cerebrovascular damage and progression resulting from Aβ deposition [73, 74]. Furthermore, trophic disbalance of the granular cells layer in DG may lead to synaptic disruptions [17, 60, 75] and weakened effectiveness of synaptic transmission to projections in CA subregions [76].

In conclusion, our stereological studies of the microvascular network in the hippocampus find both between-group effects in 12-month-old male Tg rats compared to age- and sex-matched non-Tg controls, together with subregional differences (i.e., within-group effects) in Tg rats that are not present in non-Tg. These findings include significantly lower Total L_cap_ in the DG region and 53% higher average capillary length (Total L_cap_/Total N_cap_) for total hippocampus in Tg compared to non-Tg rats. Among within-group differences in Tg rats are differences in number and length density of capillary in neuron cells layer with the most marked differences in the DG. Similarities with AD-type microcirculatory changes reported in humans suggest the TgF344-AD rat model provides a useful *in-vivo* model for enhancing understanding and pre-clinical drug discovery for future studies of the cerebral amyloid angiopathy associated with AD in humans.
